# First case of human ehrlichiosis in Mexico.

**DOI:** 10.3201/eid0503.990327

**Published:** 1999

**Authors:** R A Gongóra-Biachi, J Zavala-Velázquez, C J Castro-Sansores, P González-Martínez


### First Case of Human Ehrlichiosis in Mexico

**To the Editor:** Ehrlichiosis is a zoonotic disease transmitted to humans through the bite of infected ticks (1). The first recognized human ehrlichial infection, Sennetsu fever, was described in Japan in 1954 (2). The first case of human ehrlichiosis in the United States was recognized in 1986 and was reported in 1987 (3). The disease is caused by intracellular gram-negative bacteria of the *Ehrlichia* genus. The bacteria can be found in the monocytes and granulocytes of peripheral blood. Human monocytic ehrlichiosis is caused by *E. chafeensis,* and human granulocytic ehrlichiosis is caused by *E. equi* or *E. phagocytophilia,* which was first recognized in 1994 (4). Most cases occur between April and September, and the reservoirs are field animals such as rodents, deer, and dogs. The clinical spectrum of the disease is similar to that of other febrile illnesses; without adequate and timely treatment, approximately 5% of the patients die (5).

In the United States, more than 400 cases of serologically confirmed *E. chaffensis* infection have been documented since 1996 (6). No cases have been reported in Mexico.

In February 1997, we evaluated a 41-year-old male patient from Merida. The patient had been exposed to ticks during activity in a rural area 1 week before the onset of illness. Clinical manifestations included frequent hyperthermia, rash, myalgia, headache, anorexia, fatigue, and cough. Physical examination showed bilateral cervical lymphadenopathy, and a chest radiograph showed an interstitial bilateral infiltrate. Hematic cytometry showed thrombocytopenia of 134 x $10^3/\mu L$ and 3200 leukocytes (1440 neutrophils/$\mu L$). Hepatic transaminases were elevated, with an aspartate aminotransferase: 92 U/L (normal: 22 U/L), alanine aminotransferase: 48 U/L (normal: 18 U/L), gamma-glutamyltranspeptidase: 278 U/L (normal: 28 U/L); and globulins: 4.8 g/dL with a polyclonal pattern. No antibodies against rickettsia, dengue virus, B-19 parvovirus, or HIV were detected. A serum sample gave a positive reaction by indirect immunofluorescence assay against *E. chaffeensis* at titers of 1:64 on week 2 and 1:128 on week 3. No infected monocytes or granulocytes were observed in peripheral blood. Remission of the clinical manifestations began on week 4 and was completed on week 6.

This case indicates the existence of human ehrlichiosis in Yucatan, Mexico. Reactivity to *E. chaffeensis* suggests human monocytic ehrlichiosis; however, as antibody testing was not performed with *E. phagocytophila* or *E. equi,* the possibility of human granulocytic ehrlichiosis cannot be excluded. In any event, case reports indicate the need for deliberate search for cases. Dengue is endemic in this area of Mexico, and ehrlichiosis should be considered as a differential diagnosis.

**Renán A. Gongóra-Biachi,* Jorge Zavala-Velázquez, † Carlos José Castro-Sansores,* and Pedro González-Martínez***

*Centro de Investigaciones Regionales "Dr. Hideyo Noguchi," Mérida, Yucatán, México; and †Facultad de Medicina, Universidad Autónoma de Yucatán, Mérida, Yucatán, México

### References

1. Dumler SJ, Bakken JS. Ehrlichial diseases of humans: emerging tick-borne infections. Clin Infect Dis 1995;20:1102-10.
2. Schaffner W, Standaert SM. Ehrlichiosis—in pursuit of an emerging infection. N Engl J Med 1996;334:262-3.
3. Maeda K, Markowitz N, Hawley RC, Ristic M, Cox D, McDade JE. Human infection with *Ehrlichia canis*, a leucocytic rickettsia. N Engl J Med 1987;316:853-6.
4. Bakken JS, Dumler JS, Chen SM, Eckman MR, Van Etta LL, Walker DH. Human granulocytic ehrlichiosis in the upper midwest United States: a new species emerging? JAMA 1994;272:212-8.
5. Walker D, Raoult D, Brouqui P, Marrie T. Rickettsial diseases. In: Fauci AS, Braunwald E, Isselbacher KJ, Wilson JD, Martin JB, Kasper DL, et al., editors. Harrison's principles of internal medicine. 14th ed. New York: The McGraw-Hill Companies; 1998. p. 1045-52.
6. Walker DH, Dumler JS. Emergence of the ehrlichioses as human health problems. Emerg Infect Dis 1996;2:18-29.

### HIV-1 Subtype F in Single and Dual Infections in Puerto Rico: A Potential Sentinel Site for Monitoring Novel Genetic HIV Variants in North America

**To the Editor:** Although international efforts to systematically collect, characterize, and classify HIV isolates from around the world have increased considerably, data on HIV-1 genetic variations in Puerto Rico are limited. This island (population 3.7 million) has one of the highest

